# The survival of multi-drug resistant bacteria on raw Douglas fir material

**DOI:** 10.1038/s41598-024-53983-4

**Published:** 2024-02-12

**Authors:** A. Taisne, F. Aviat, M. Essono Mintsa, C. Belloncle, H. Pailhoriès

**Affiliations:** 1 Laboratoire de Bactériologie-Hygiène, Centre Hospitalier Universitaire, 4 rue Larrey, 49933 Angers cedex, France; 2 Your ResearcH-Bio-Scientific, 307 la Gauterie, 44430 Le Landreau, France; 3 Laboratoire Innovation Matériau Bois Habitat (LIMBHA), Ecole Supérieure du Bois, 7 rue Christian Pauc, 44000 Nantes, France; 4 Laboratoire HIFIH, UPRES EA3859, SFR 4208, Université d’Angers, Angers, France

**Keywords:** Bacteria, Environmental microbiology

## Abstract

In today’s age of ecological transition, the use of materials such as renewable wood in construction is particularly relevant, but also a challenge in the healthcare sector where the hygiene dimension also comes into play. In this study we have investigated the survival of multi-resistant bacteria commonly responsible for healthcare-associated infections (HAIs) (ESBL-positive *Klebsiella pneumoniae* and glycopeptide-resistant *Enterococcus faecalis*) on two different types of wood (Douglas fir : *Pseudotsuga menziesii* and Maritime Pine : *Pinus pinaster*) compared to other materials (smooth: stainless steel and rough: pumice stone) and the effect of a disinfection protocol on the bacterial survival *on Pseudotsuga menziesii*. Approximately 10^8^ bacteria were inoculated on each material and bacterial survival was observed over several days (D0, D1, D2, D3, D6, D7 and D15). Each analysis was performed in triplicate for each time and material. The results show an important reduction of the bacterial inoculum for *Klebsiella pneumoniae* and *Enterococcus faecalis* on Douglas fir, in contrast with the results obtained on maritime pine, stainless steel and pumice stone. No bacterial survival was detected on Douglas fir after application of a hospital disinfection protocol. These different results show that wood may have a place in the future of healthcare construction. Further studies would be interesting to better understand the different properties of wood.

## Introduction

Building materials vary in nature and can therefore respond to the mechanical, economic and, in some cases, public health needs of the structures for which they are intended. Building materials used in the healthcare environment are required to have certain qualities, particularly in terms of hygiene standards.

In France, the NF ISO 14644 standard for cleanrooms and related controlled environments lists the requirements for the construction of cleanrooms, but does not specify the technological and contractual means of meeting these requirements^1^. The standard also covers the selection of materials for the construction of cleanrooms, specifying that materials chosen should be easily and frequently disinfected, with smooth, non-porous or non-rough surfaces to limit the risk of retention of chemical particles and contamination by micro-organisms. Thus, porous surfaces such as wood are not permitted in medical environments, although work has been carried out for years on the hygienic properties of wooden surfaces. Wood is one of the materials used in the construction and interior design of various structures and is still used in various forms (solid wood, plywood, panels, etc.) in medical facilities around the world.

In addition to its benefits as renewable material known for its low ecological impact, wooden surfaces have other qualities that are essential in medical environments. Indeed, when used for interior design, several studies showed that wood have an impact on psychological, physical and emotional well-being, this being known as the concept of biophilic design^2^. The use of wood in construction sector has been shown to influence 4 out of the 5 human senses: hearing, touch, sight and olfaction. Moreover, it has been shown to have an impact on physiological parameters such as improve heart rate, blood pressure, nervous system activity and even emotions in patients waiting in a wooden waiting room. Wood can also improve the acoustic properties of a room and regulate humidity levels^3,4^. Despite its advantages, in healthcare buildings, wood is still considered "unhygienic" and “difficult to clean” due to its anatomical properties (porosity and organic composition)^5^. In the healthcare sector, hygiene is a priority, as it determines the safety of patient stays by limiting the risk of nosocomial infections.

Several papers have focused on hygienic properties of wooden surfaces, in particular by assessing the survival of microorganisms responsible for HAI and have shown some antibacterial properties of wood, leading to consider this material as an alternative for healthcare buildings^5–8^. Pailhoriès et al. tested and showed the antimicrobial activity of oak on a panel of *S. aureus* with different resistance patterns to antibiotics^7^. Then, a study of Chen et al. has shown an effect of oak on the survival of bacteria classically responsible for HAIs (*Acinetobacter baumannii*, *Enterococcus faecalis*, *Klebsiella pneumoniae*, *Staphylococcus aureus*) compared to aluminium, polycarbonate and stainless steel^8^. Munir et al. proved wood to be less exposed to the development of *S. aureus* bacterial biofilm than other materials such as melamine^9^. However, some studies also highlight that this antibacterial effect of raw wood material can vary according to the wood species. Indeed, some results suggest that Douglas fir has greater antibacterial properties than other wood species. Furthermore, the effect of wood treatment on this properties, such as varnishing or disinfection, has not been evaluated^9^.

The suggested antibacterial activity of wooden surfaces could be a benefit in the fight against bacteria responsible for healthcare-associated infections (HAI) in healthcare environment. Indeed, HAI represent a major health issue for the years to come, with multiple and global impact. In 2017, in France, HAI have affected 1 out of 20 patients and were responsible for 4 000 deaths^10^. In Europe, 2.6 million deaths due to HAIs are reported each year^11^. In the United States, HAIs accounted for 3.2% of hospitalisations in 2015^12^. In 2011 a WHO report revealed that the global annual European financial loss related to these infections was 7 billion euros^13^. Among infectious agents involved, multi-drug resistant (MDR) bacteria pose a large public health problem. The WHO published a list of “priority pathogens” for the research and development of new antibiotics, including carbapenem-resistant *Acinetobacter baumannii*, carbapenem-resistant *Pseudomonas aeruginosa* and carbapenem-resistant and ESBL-producing Enterobacteriaceae among the most critical pathogens of this list^14^. Among the means used to reduce the incidence of HAI, one privileged axis has been to limit the risk factors of bacterial acquisition. In recent years, part of the campaign against HAIs has been based on hospital hygiene recommendations and education program for healthcare workers^15^, such as good practices of disinfection for patient rooms or washing hand^16^, using sterile single-use equipment^17^. Indeed, the immediate hospital environment of patients in the care setting (rooms, waiting rooms, etc.) is one potential vector of microorganisms by offering support^16,18–21^.

The aim of this study is (i) to assess the persistence of bacteria over time on different materials, as 316 TI stainless steel, pumice stone, two different wooden species including Douglas fir (*Pseudotsuga menziesii*), maritime pine (*Pinus pinaster*) and varnished Douglas fir, under different conditions and (ii) to test the impact of wood disinfection of Douglas fir as assessment of the bacterial survival.

## Material & methods

### Material preparation

Five materials were tested in this study to represent different physico-chemical properties and to compare their anti-bacterial properties: 316 TI stainless steel, pumice stone, two different species of wood including Douglas fir (*Pseudotsuga menziesii*), maritime pine (*Pinus pinaster*) and varnished Douglas fir. The Douglas fir and maritime pine used originated from a radius of 200 km around Nantes (Pays de la Loire, France). The trees were air-dried for at least 6 months, then artificially dried and sawn into blocks at the ESB (Higher School of Wood and Biosourced Materials) using a mobile saw. These blocks are then stripped of sapwood and sawn to a variable thickness of 2.5 to 3.5 mm using a radial saw (Altendorf F45, Minden, Germany or Bosch GCM). In order to obtain the sample discs, a random cross-sectional removal from 4 slices with a punch (Facom, 10 mm) has been performed. Afterwards, the wood has been stored in a climate chamber at 22 °C and 50% relative humidity, to obtain a moisture content of 12%. A group of Douglas fir discs have been varnished with two coats of a silver nitrate-water suspension (100 g/m^2^ by brush).

### Bacterial strains

Two bacterial strains were used in this study: Gram negative *Klebsiella pneumoniae* American Type Culture Collection (ATCC) 700603 producing extended-spectrum beta-lactamase (ESBL) and Gram positive *Enterococcus faecalis* ATCC 51299 presenting the *vanB* gene inducing glycopeptide resistance. The cultures were grown on Columbia agar with 5% sheep blood (Bio-Rad, Marnes-La-Coquette, France) and incubated for 24 h at 35 ± 2 °C.

### Survival assay

The protocol used in this study is adapted from the protocol previously used by Chen et al.^8^. Briefly, for each bacterium, a bacterial colony has been inoculated into Brain Heart Infusion broth (BioMérieux, Marcy l’Etoile, France). Incubation was performed for 12 to 18 h at 35 ± 2 °C. A 3 ml aliquot of this culture was then centrifuged (12,000*g*, 5 min), the supernatant removed, and the pellet washed twice with 3 ml of 0.9% sodium chloride solution (Aguettant, France).

A 20 µl inoculum of the resulting bacterial solution (approximately 10^8^ Colony Forming Units (CFU)) has been deposited on each material sample disc including stainless steel, pumice stone, raw Douglas fir, varnished Douglas fir; raw maritime pine and Douglas fir which had previously been disinfected daily for 30 consecutive days with an alcohol-free disinfectant detergent foam (ECOSEPTOL FOAM, Franklab, Montigny Le Bretonneux, France). The discs were placed in covered petri dishes at ambient temperature. The enumeration of the initial bacterial suspensions was performed by tenfold serial dilutions, inoculated on blood agar plates. The survival assays were performed in three consecutive experiments, one comparing stainless steel, pumice stone, Douglas fir and varnished Douglas fir (Assay 1), one comparing maritime pine and stainless steel (Assay 2) and a last one comparing Douglas fir and disinfected Douglas fir (Assay 3).

### Disinfection protocol

To test the impact of wood disinfection, 42 samples of Douglas fir were disinfected daily for 30 consecutive days with an alcohol-free disinfectant detergent foam (ECOSEPTOL FOAM, Franklab, Montigny Le Bretonneux, France) used in the medical biology laboratory of Angers University Hospital for daily cleaning surfaces. The surface of the samples was completely covered with the product. The samples were then dried in the open air.

### Bacterial counts

For each daily test (Day 0, Day 1, Day 2, Day 3, Day 6, Day 7 and Day 15), bacterial counts were performed in triplicate using three specimens of each material. Briefly, these discs were shaken for 30 s at a frequency of 30 Hz in 4 mL of NaCl 0.9% with a ball mill system (MM 400, Retsch, Haan, Germany). This ball mill system procedure consisted in shaking discs at 30 Hz with 5 mm inox beads, for a best bacterial retrieval. The resulting solution underwent bacterial numeration by plating 10 µl of tenfold serial dilutions on 5% sheep blood Columbia agar plates. The bacterial counts were determined after 24 h at 35 ± 2 °C. The bacterial counts considered for the study correspond to the average number of CFU present on the triplicates per material at each time.

### Statistical analysis

For statistical analysis, the Kruskal Wallis test with Dunn's multiple comparison is used when comparing 4 materials, and the Wilcoxon-Mann–Whitney test when comparing 2 materials. The analysis is performed with the XLSTAT software (Addinsoft, Paris). For the statistical analysis and figures, the absence of CFU was converted to 1 for the purpose of the analysis.

In order to present the evolution of the colonisation of the different materials in a simple and visual way, we chose to use the BoxPlot representation, which also allows us to visually observe the differences between the materials.

### Plant collection

No plant has been collected in this study. The wood used was cut from commercially harvested logs. It was purchased from a sawmill in the Pays de la Loire region of France.

## Results

### Bacterial survival on different surfaces

**Figure 1:**
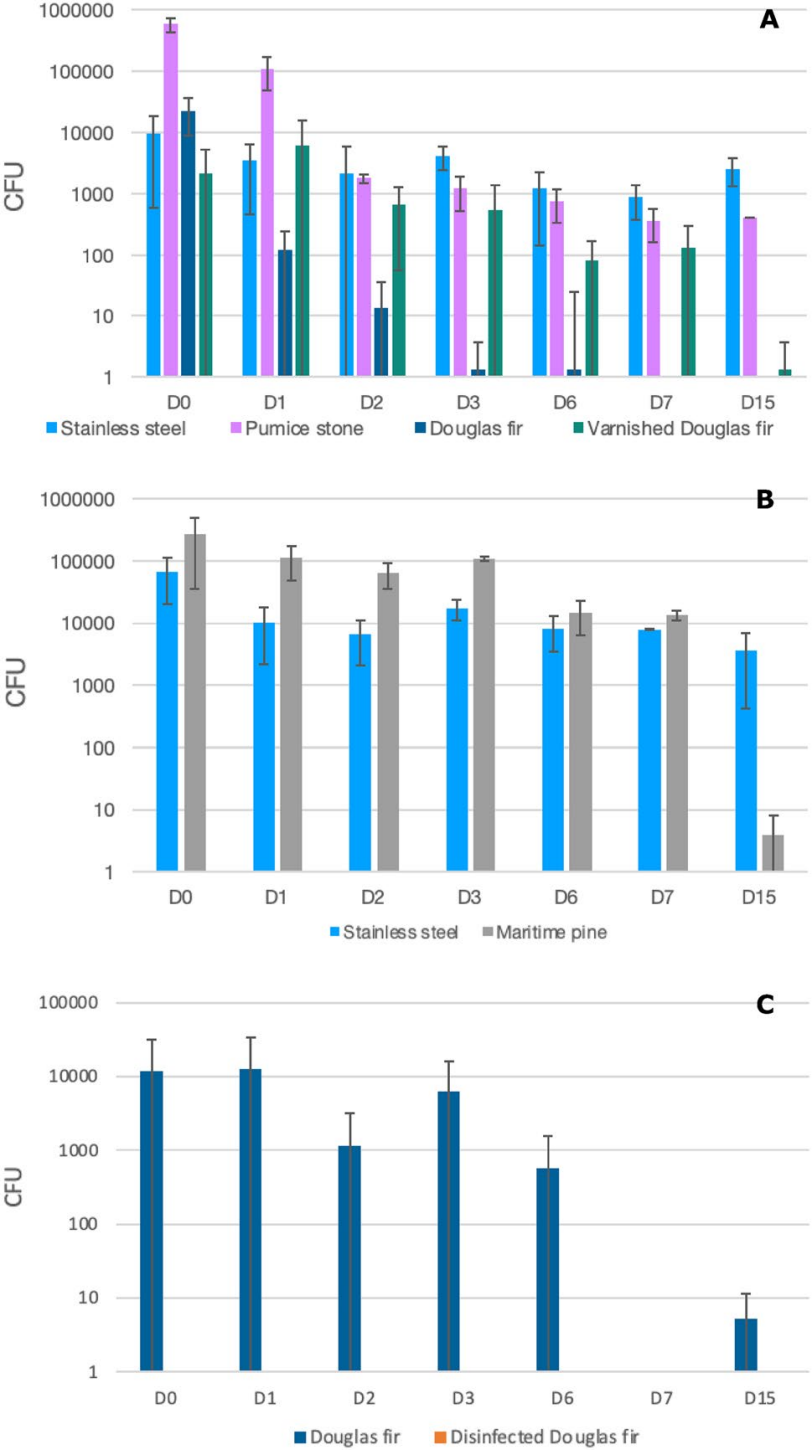
Bacterial counts of *Enterococcus faecalis* ATCC 51,299 (in colony forming units (CFU)) recovered from stainless steel, pumice stone, Douglas fir and varnished Douglas fir (Assay 1) (**A**), on stainless steel and maritime pine (Assay 2) (**B**), and on Douglas fir and disinfected Douglas fir (Assay 3) (**C**) over 15 days.

**Figure 2:**
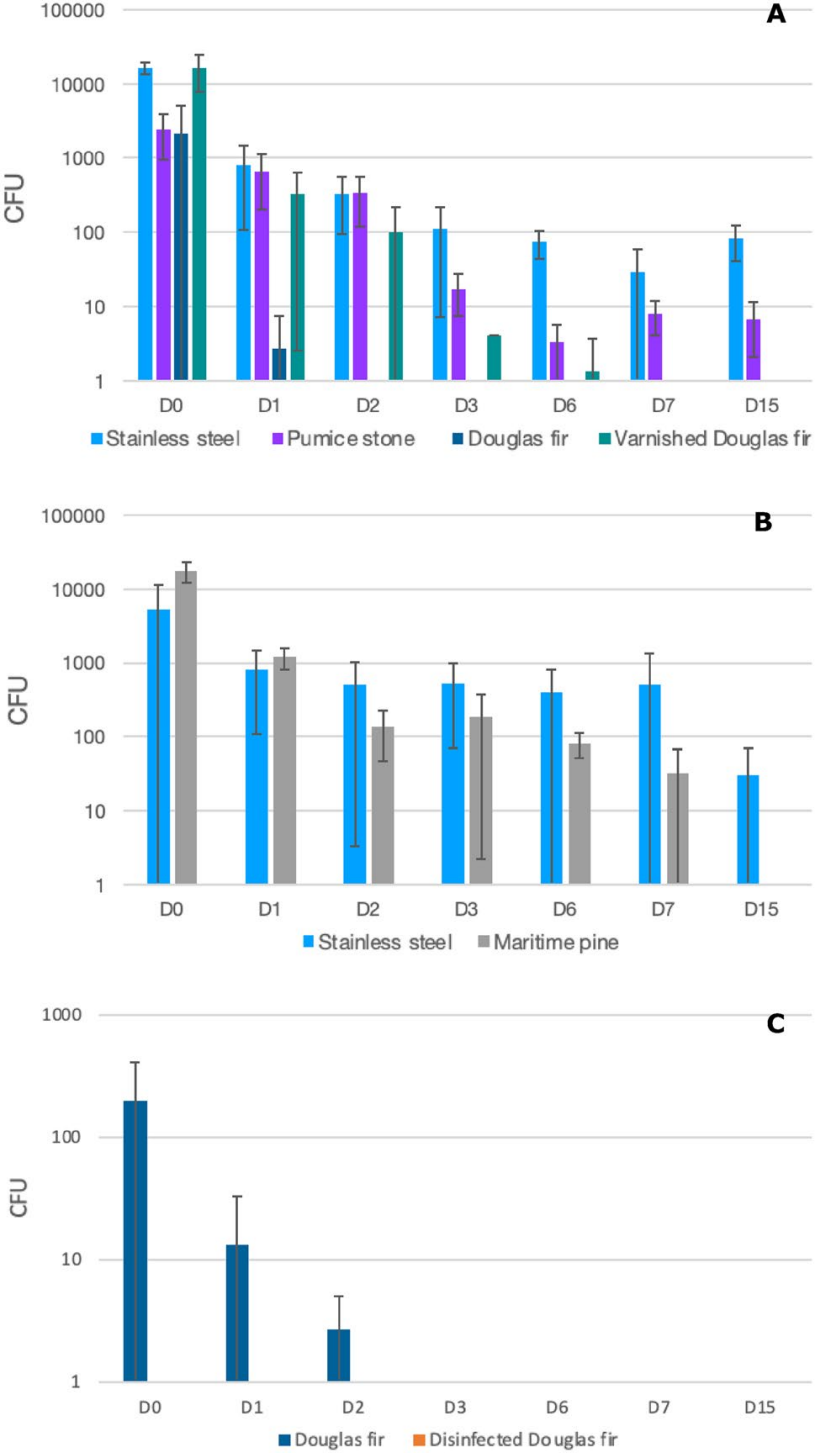
Bacterial counts of *Klebsiella pneumoniae* ATCC 700,603 recovered from stainless steel, pumice stone, Douglas fir and varnished Douglas fir (Assay 1) (**A**), on stainless steel and maritime pine (Assay 2) (**B**), and on Douglas fir and disinfected Douglas fir (Assay 3) (**C**) over 15 days.

On the first assay for stainless steel (Assay 1), the amount of *E. faecalis* decreased in the first 24 h from 9.47 × 10^3^ ± 8.88 × 10^3^ to 3,47 × 10^3^ ± 3.00 × 10^3^ CFUs (a reduction of 0.44 log_10_ CFUs), and then reduced slightly to 2.53 × 10^3^ ± 1.22 × 10^3^ CFUs to day 15 (Table 1) (Fig. 1A). The survival assay performed on the second assay (Assay 2), confirmed this survival scheme of *E. faecalis* on stainless steel with a decrease of 0.82 log_10_ CFUs in the first 24 h (from 6.67 × 10^4^ ± 4.62 × 10^4^ to 1.01 × 10^4^ ± 8.01 × 10^3^ CFUs), and then a slight reduction to 3.60 × 10^3^ ± 3.17 × 10^3^ CFUs to day 15 (Table 1) (Fig. 1B). Concerning *K. pneumoniae*, a decrease from 1.65 × 10^4^ ± 3.06 × 10^3^ to 8.00 × 10^2^ ± 6.93 × 10^2^ CFUs over the first 24 h (a reduction over this period of 1,31 log_10_ CFUs), then decreasing to 8.13 × 10^1^ ± 4.09 × 10^1^ CFUs on day 15 (Table 2) (Fig. 2A). Again, the bacterial survival kinetic was confirmed on the assay 2 for this material, with a decrease of *K. pneumoniae* of 1 log_10_ CFUs over the first 24 h (8.00 × 10^3^ ± 6.11 × 10^3^ to 8.00 × 10^2^ ± 6.93 × 10^2^ CFUs), then a decrease to 3.07 × 10^1^ ± 4.01 × 10^1^ CFUs on day 15 (Table 2) (Fig. 2B).

**Table 1:** Average bacterial count in CFU (colony forming unit) of *Enterococcus faecalis* on several materials over time.

*Enterococcus faecalis*, ATCC 51299	Materials	Inoculum (CFU/20 µL)	CFU						
			D0	D1	D2	D3	D6	D7	D15
Assay 1	Stainless steel	3.20E + 08	9.47E + 03 ± 8.88E + 03	3.47E + 03 ± 3.00E + 03	2.13E + 03 ± 3.70E + 03	4.09E + 03 ± 1.65E + 03	1.20E + 03 ± 1.06E + 03	8.80E + 02 ± 5.00E + 02	2.53E + 03 ± 1.22E + 03
	Pumice stone		5.87E + 05 ± 1.51E + 05	1.09E + 05 ± 6.06E + 04	1.79E + 03 ± 3.00E + 02	1.20E + 03 ± 6.93E + 02	7.47E + 02 ± 4.20E + 02	3.60E + 02 ± 2.00E + 02	4.00E + 02 ± 0
	Douglas fir		2.27E + 04 ± 1.40E + 04	1.20E + 02 ± 1.20E + 02	1.33E + 01 ± 2.31E + 01	1.33E + 00 ± 2.31E + 00	1.33E + 01 ± 2.31E + 01	Absence	Absence
	Varnished Douglas fir		2.13E + 03 ± 3.03E + 03	6.00E + 03 ± 9.70E + 03	6.67E + 02 ± 6.11E + 02	5.47E + 02 ± 8.45E + 02	8.13E + 01 ± 8.64E + 01	1.32E + 02 ± 1.60E + 02	1.33E + 00 ± 2.31E + 00
Assay 2	Stainless steel	1.40E + 08	6.67E + 04 ± 4.62E + 04	1.01E + 04 ± 8.01E + 03	6.67E + 03 ± 4.62E + 03	1.73E + 04 ± 6.11E + 03	8.13E + 03 ± 4.60E + 03	7.87E + 03 ± 1.62E + 02	3.60E + 03 ± 3.17E + 03
	Maritime pine		2.67E + 05 ± 2.31E + 05	1.12E + 05 ± 6.44E + 04	6.40E + 04 ± 2.88E + 04	1.07E + 05 ± 8.33E + 03	1.47E + 04 ± 8.33E + 03	1.33E + 04 ± 2.31E + 03	4.00E + 00 ± 4.00E + 00
Assay 3	Douglas fir	9.20E + 08	1.19E + 04 ± 1.95E + 04	1.27E + 04 ± 2.13E + 04	1.17E + 03 ± 2.00E + 03	6.13E + 03 ± 9.60E + 03	5.73E + 02 ± 9.93E + 02	Absence	5.33E + 00 ± 6.11E + 00
	Disinfected Douglas fir		Absence	Absence	Absence	Absence	Absence	Absence	Absence

**Table 2:** Average bacterial count in CFU (colony forming unit) of *Klebsiella pneumoniae* on several materials over time.

*Klebsiella pneumoniae*, ATCC 700603	Materials	Inoculum (CFU/20 µL)	CFU						
			D0	D1	D2	D3	D6	D7	D15
Assay 1	Stainless steel	2.60E + 08	1.65E + 04 ± 3.06E + 03	8.00E + 02 ± 6.93E + 02	3.24E + 02 ± 2.30E + 02	1.11E + 02 ± 1.04E + 02	1.11E + 02 ± 3.00E + 01	2.93E + 01 ± 3.00E + 01	8.13E + 01 ± 4.09E + 01
	Pumice stone		2.40E + 03 ± 1.44E + 03	6.67E + 02 ± 4.62E + 02	3.35E + 02 ± 2.15E + 02	1.73E + 01 ± 1.01E + 01	5.33E + 00 ± 2.31E + 00	8.00E + 00 ± 4.00E + 00	6.67E + 00 ± 4.62E + 00
	Douglas fir		2.13E + 03 ± 3.00E + 03	2.67E + 00 ± 4.62E + 00	Absence	Absence	Absence	Absence	Absence
	Varnished Douglas fir		1.61E + 04 ± 8.32E + 03	3.20E + 02 ± 3.17E + 02	1.01E + 02 ± 1.19E + 02	4.00E + 00 ± 0	1.33E + 00 ± 2.31E + 00	Absence	Absence
Assay 2	Stainless steel	5.40E + 08	8.00E + 03 ± 6.11E + 03	8.00E + 02 ± 6.93E + 02	5.07E + 02 ± 5.03E + 02	5.20E + 02 ± 4.51E + 02	4.00E + 02 ± 4.00E + 02	5.07E + 02 ± 8.43E + 02	3.07E + 01 ± 4.01E + 01
	Maritime pine		1.75E + 04 ± 5.33E + 03	1.20E + 03 ± 4.00E + 02	1.37E + 02 ± 8.99E + 01	1.85E + 02 ± 1.83E + 02	8.13E + 01 ± 2.95E + 01	3.20E + 01 ± 3.67E + 01	Absence
Assay 3	Douglas fir	6.80E + 08	2.00E + 02 ± 2.12E + 02	1.33E + 01 ± 1.97E + 01	2.67E + 00 ± 2.31E + 00	Absence	Absence	Absence	Absence
	Disinfected Douglas fir		Absence	Absence	Absence	Absence	Absence	Absence	Absence

On pumice stone, the amount of *E. faecalis* decreased in the first 24 h from 5.87 × 10^5^ ± 1.51 × 10^5^ to 1.09 × 10^5^ ± 6.09 × 10^4^ CFUs (a reduction of 0.72 log_10_), then decreasing up to 4.00 × 10^2^ ± 0 CFUs to day 15 (Table 1) (Fig. 1A). Concerning *K. pneumoniae*, a decrease from 2.40 × 10^3^ ± 1.44 × 10^3^ o 6.67 × 10^2^ ± 4.62 × 10^2^ CFUs was observed in the first 24 h (a reduction over this period of 0.56 log_10_), then decreasing to 6.67 ± 4.62 CFUs on day 15 (Table 2) (Fig. 2A).

On the Douglas fir (Assay 1), the amount of *E. faecalis* decreased over the first 24 h from 2.27 × 10^4^ ± 1.40 × 10^4^ to 1.20 × 10^2^ ± 1.20 × 10^2^ CFUs, (a reduction of 2.28 log_10_), then reduced to the absence of viable colonies from day 7 (Table 1) (Fig. 1A). The survival assay performed on the third assay (Assay 3), not confirmed exactly this survival scheme of *E. faecalis* on Douglas fir with an increase of 0.03 log_10_ CFUs in the first 24 h (from 1.19 × 10^4^ ± 1.95 × 10^4^ to 1.27 × 10^4^ ± 2.13 × 10^4^ CFUs), and then reduced to the absence of viable colonies from day 7 with a little rebound on day 15 with 5.33 ± 6.11 CFUs. (Table 1) (Fig. 1C). Regarding *K. pneumoniae*, a decline from 2.13 × 10^3^ ± 3.00 × 10^3^ to 2.67 ± 4.62 CFUs was noted over the first 24 h (a reduction over this period of 2.90 log_10_), then decreasing to the absence of colony from day 2 (Table 2) (Fig. 2A). The bacterial survival kinetic was confirmed on the assay 3 for this material, with a decrease of *K. pneumoniae* of 1.18 log_10_ CFUs over the first 24 h (2.00 × 10^2^ ± 2.12 × 10^2^ to 1,33 × 10^1^ ± 1.97 × 10^1^ CFUs), then decreasing to the absence of colony from day 3 (Table 2) (Fig. 2C).

On the varnished Douglas fir, the amount of *E. faecalis* increased in the first 24 h from 2.13 × 10^3^ ± 3.03 × 10^3^ to 6.00 × 10^3^ ± 9.70 × 10^3^ CFUs (an increase of 0.45 log_10_), then it decreased to 1.33 CFUs to day 15 (Table 1) (Fig. 1A). The decline of *K. pneumoniae* in this same material was from 1.61 × 10^4^ ± 8.32 × 10^3^ to 3.20 × 10^2^ ± 3.17 × 10^2^ CFUs 24 h (a reduction over this period of 1.70 log_10_), then it decreased to the absence of colony from day 7 (Table 2) (Fig. 2A).

On maritime pine, the amount of *E. faecalis* reduced in the first 24 h from 2.67 × 10^5^ ± 2.31 × 10^5^ to 1.12 × 10^5^ ± 6.44 × 10^4^ CFUs (a reduction of 0.38 log_10_), then it decreased to 4 ± 4 CFUs at day 15 (Table 1) (Fig. 1B). Regarding *K. pneumoniae*, a reduction from 1.75 × 10^4^ ± 5.33 × 10^3^ to 1.20 × 10^3^ ± 4.00 × 10^2^ CFUs was observed in the first 24 h (a reduction over this period of 1.16 log_10_), then decreasing to the absence of colony on day 15 (Table 2) (Fig. 2B).

The distribution of viable bacteria counts for *E. faecalis* and *K. pneumoniae* from day 0 to day 15 was significantly different between raw Douglas fir and stainless steel (p = 0.003 for *E. faecalis* and p = 0.005 for *K. pneumoniae*; Kruskal Wallis test with Dunn's multiple comparison) and pumice stone (p = 0.017 for *E. faecalis* and p = 0.031 for *K. pneumoniae*; Kruskal Wallis test with Dunn's multiple comparison) in Assay 1 (Figs. 1 and 2).

No significant difference was noticed in the distribution of viable bacteria counts for *E. faecalis* and *K. pneumoniae* between the other materials (Figs. 1 and 2) in Assay 1 and 2.

### Distribution of viable bacteria after daily disinfection

After thirty days of daily disinfection on Douglas fir (Assay 3), no bacteria were counted during the entire study period (day 0 to day 15) for both *Enterococcus faecalis* and *Klebsiella pneumoniae* (Tables 1 and 2) (Figs. 1C and 2C). In the absence of prior disinfection, both strains colonised Douglas fir, with *E. faecalis* colonising for longer and at higher levels (1.19 × 10^4^ ± 1.95 × 10^4^ CFUs at day 0 to 5.33 ± 6.11 CFUs at day 15) than *K. pneumoniae* (2.00 × 10^2^ ± 2.12 × 10^2^ CFUs at day 0 to none at day 3). The distribution of viable *E. faecalis* counts from day 0 to day 15 is significantly different between raw Douglas fir and disinfected Douglas fir (p = 0.005; Wilcoxon-Mann–Whitney test). However, for *K. pneumoniae* there was no significant difference (p = 0.192 Wilcoxon-Mann–Whitney test) between these two materials (Figs. 1 and 2). This absence of statistical difference over the 15 days for *K. pneumoniae* is due to the absence of viable bacteria on Douglas fir after the day 2. Thus, statistical analysis was performed from Day 0 to Day 2, and there a significant difference was observed between the two materials (p = 0.0018; Wilcoxon-Mann–Whitney test).

## Discussion

Environmental management is a key issue in the healthcare sector. Indeed, the environment can be a reservoir for a wide range of pathogens, including multidrug-resistant bacteria potentially responsible for HAI. The aim of this study was to examine the survival of MDR bacteria (ESBL-producing *K. pneumoniae*, *vanB*-carrying *E. faecalis*) on raw wood compared with a smooth material widely used in healthcare facilities synthetic (stainless steel), rough material (pumice stone) and treated wood (varnished and disinfected).

This study indicates that bacterial survival decreases more rapidly on Douglas fir compared to stainless steel and pumice stone due to wooden porosity whereas stainless steel is smooth surface which is a favorable condition for bacteria survival, suggesting natural antibacterial properties. Several studies in the literature point in this direction. The resin extract of Douglas fir has shown antimicrobial activity against certain anaerobic bacteria (*Actinomyces bovis*, *Clostridium perfringens*), explained by the presence of terpenes including resin acids^22^. The essential oil obtained from twigs, leaves, bark and needles of Douglas fir also showed bacteriostatic activity on several bacteria potentially responsible of infections such as *Staphylococcus aureus*, *Proteus mirabilis*, *Serratia marcescens*^23^. However, in these last studies, some extracts of Douglas fir have been tested rather than the raw wood used in our study. Testing the raw material rather than the extractives, allow to give a global point of view of antibacterial activity of raw wood, and thus its interest in healthcare buildings. Indeed, when tested in an isolated way, the extractives are in much higher concentrations than in raw wood. We also observe in this study a slight difference between the survival time of Gram-negative bacteria and Gram-positive bacteria: Gram-positive bacteria being associated with a longer survival. Another study has already highlighted this difference, with a reduced antibacterial effect of Douglas fir essential oils on *Staphylococcus aureus* and *Bacillus cereus* (Gram-positive) compared with *Pseudomonas aeruginosa* (Gram-negative)^24^, this finding has not yet been explained, and hypotheses need to be developed. These results of Douglas fir on bacterial survival, and such differences of activity depending on the bacterial species should be further investigated.

In this study, the antibacterial activity of maritime pine has also been tested. Several studies on the antibacterial properties of extracts of maritime pine^25,26^. It has been shown an antibacterial activity on Gram-positive bacteria with little or no effect on Gram-negative bacteria. This difference in results can be explained by the difference in phenolic acid and flavonoid content between a piece of raw wood and a purified extract. In fact, we can make the hypothesis of low levels of these extracts in the wood samples tested here, which would impact the antibacterial properties. Another studies show the antibacterial properties of oak compared to other materials, including stainless steel, on four bacteria, two of which were identical to those used in our study^8^ and a significantly greater decrease in *Staphylococcus aureus* colonisation over time on Douglas fir compared to oak and poplar^9^. So, it can be said the antibacterial properties of wood are timber-specific (Douglas fir, oak *versus* maritime pine), showing that the antibacterial action of certain species is not only due to the mechanical properties of the wood, but also to its molecular composition, its pH level, as several studies have shown^5,27^. This leaves a wide field of research for the coming years, since in France alone there are about 190 different species of wood, divided into 7 main species (oak, beech, chestnut, maritime pine, Scots pine, spruce and fir).

Two conditions have been tested on Douglas fir material: antibacterial coating and iterative disinfection. Douglas fir with an antibacterial coating showed no difference in bacterial survival compared to other materials, the coating can moderate the chemical and physical antibacterial activities of wood by affecting its surface structure, it can be said that this contributes to its lower effectiveness compared to raw Douglas fir *versus* stainless steel. In fact, several studies^9,28,29^ show that the porosity of wood has an influence on its antibacterial properties, in particular by trapping bacteria deep down in an unsuitable environment for their survival. Similarly, Douglas fir shows a significant reduction in bacterial survival compared to pumice, a very porous material, which supports the fact that porosity plays a role in the antibacterial properties of wood, but is not the only one. The synergy of several mechanisms is responsible for this antibacterial effect. Furthermore, the study of the disinfected wood shown a remanent effect with an absorbing phenomenon of the disinfectant. Actually, there is no colonisation of the wood after daily disinfection, similar to that used in health institutions, even with a high bacterial inoculum deposited on to the surface. The differences observed between disinfected and non-disinfected Douglas fir highlights the strong impact of such disinfection of the material on viable bacteria. The impact of such iterative disinfections on the physical and chemical properties of raw wood should be better characterized before recommending such protocols on wooden materials.

This study has certain biases, such as the difficulty to recover the bacteria colonising the surface of the materials after inoculation, and which gives only a partial idea if bacteria colonizing wooden material. Different systems were used to recover bacteria from wood surfaces, such as sponge contact method^30^, brushing recovery method^31^, orbital shaking^32^ or destructive methods^33^. The ball mill system method used in this study has been chosen for the retrieval of bacteria in the surface, reflecting the possible contamination by contact. However, a destructive method would maybe have allowed a better picture of wood bacterial contamination and would be investigated in further studies. Also, the preparation process used here for the wood may have an influence on antimicrobial properties, and other preparation procedures should be tested. Furthermore, the surface and thickness of the disinfected discs are far from the conditions in the construction sector and make it difficult to understand/identify the effect of disinfection in real conditions. Next step will be testing bacterial survival on real surfaces.

## Conclusion

The results show that Douglas fir has interesting natural antibacterial properties, particularly for the design of environments in the healthcare sector, indeed healthcare facilities receive a "sensitive" public, so hygiene is essential in this environment. Furthermore, several studies show that the use of natural materials such as wood in the direct environment of the patient allows an improvement in his well-being. It can play a role in the length of his hospital stay^3,4,34^. Further studies are needed to understand the differences in antibacterial activity between wooden species. Indeed, the effect of wood origin, wood aging, chemical profile (quality and quantity), pH level, modified surface is still poorly understood. Knowledge of the molecular composition of wooden species is one of the directions to be explored. The study of a specific disinfection protocol adapted to the wooden material could also be important to bring natural elements belong to biophilic design in these specific environments for calming down patients, their families and medical support.

### Supplementary Information

Supplementary Tables.

## Data Availability

All data generated or analysed during this study are included in the published article (and Supplementary Information files).
