# Supplementary material for: The survival of multi-drug resistant bacteria on raw Douglas fir material
Source: Sci Rep. 2024 Feb 12;14:3546. doi: 10.1038/s41598-024-53983-4 (PMC10861437; doi:10.1038/s41598-024-53983-4)
Supplement: Supplementary file 1 — Supplementary Tables. [file 41598_2024_53983_MOESM1_ESM.doc]

**Supplemental tables**

Table S1 : Statistical analysis of bacterial counts of *Enterococcus faecalis* ATCC 51299 (in Colony Forming Units (CFU)) recovered from stainless steel, pumice stone, Douglas fir and varnished Douglas fir (Assay 1), on stainless steel and maritime pine (Assay 2), and on Douglas fir and disinfected Douglas fir (Assay 3) over 15 days.

|  |  | p-value | | | |
| --- | --- | --- | --- | --- | --- |
| *Enterococcus faecalis* ATCC 51299 | | D0 to D15 | | | |
|  | Materials | Pumice stone | Douglas fir | | Varnished Douglas fir |
| Assay 1 | Stainless steel | 0.569 | **0.003** | | 0.082 |
|  | Pumice stone |  | **0.017** | | 0.242 |
|  | Douglas fir |  |  | | 0.223 |
|  | Materials | Stainless steel | | | |
| Assay 2 | Maritime pine | 0.128 | | | |
|  | | D0 to D2 | | D0 to D15 | |
|  | Materials | Douglas fir | | Douglas fir | |
| Assay 3 | Disinfected Douglas fir | **0.0018** | | **0.005** | |

Table S2 : Statistical analysis of bacterial counts of *Klebsiella pneumoniae* ATCC 700603 (in Colony Forming Units (CFU)) recovered from stainless steel, pumice stone, Douglas fir and varnished Douglas fir (Assay 1), on stainless steel and maritime pine (Assay 2), and on Douglas fir and disinfected Douglas fir (Assay 3) over 15 days.

|  |  | p-value | | | |
| --- | --- | --- | --- | --- | --- |
| *Klebsiella pneumoniae* ATCC 700603 | | D0 to D15 | | | |
|  | Materials | Pumice stone | Douglas fir | | Varnished Douglas fir |
| Assay 1 | Stainless steel | 0.513 | **0.005** | | 0.116 |
|  | Pumice stone |  | **0.031** | | 0.359 |
|  | Douglas fir |  |  | | 0.213 |
|  | Materials | Stainless steel | | | |
| Assay 2 | Maritime pine | 0.364 | | | |
|  | | D0 to D2 | | D0 to D15 | |
|  | Materials | Douglas fir | | Douglas fir | |
| Assay 3 | Disinfected Douglas fir | **0.0018** | | 0.192 | |

Table S3: Bacterial counts (triplicate values) in CFU (colony forming unit) of *Enterococcus faecalis* recovered from stainless steel, pumice stone, Douglas fir and varnished Douglas fir (Assay 1), on stainless steel and maritime pine (Assay 2), and on Douglas fir and disinfected Douglas fir (Assay 3) over 15 days.

| *Enterococcus faecalis*  ATCC 51299 | | CFUs | | | | | | | | | | | | | | | | | | | | |
| --- | --- | --- | --- | --- | --- | --- | --- | --- | --- | --- | --- | --- | --- | --- | --- | --- | --- | --- | --- | --- | --- | --- |
|  |  | D0 | | | D1 | | | D2 | | | D3 | | | D6 | | | D7 | | | D15 | | |
|  | Materials | CFU_1_ | CFU_2_ | CFU_3_ | CFU_1_ | CFU_2_ | CFU_3_ | CFU_1_ | CFU_2_ | CFU_3_ | CFU_1_ | CFU_2_ | CFU_3_ | CFU_1_ | CFU_2_ | CFU_3_ | CFU_1_ | CFU_2_ | CFU_3_ | CFU_1_ | CFU_2_ | CFU_3_ |
| Assay 1 | Stainless steel | 1.76x10^4^ | 1.08x10^4^ | 0 | 3.06x10^3^ | 6.40x10^3^ | 4.00x10^2^ | 0 | 0 | 6.4x10^3^ | 6.00x10^3^ | 3.20x10^3^ | 3.08x10^3^ | 1.60x10^3^ | 2.00x10^3^ | 0 | 3.20x10^2^ | 1.04x10^3^ | 1.28x10^3^ | 2.80x10^3^ | 3.60x10^3^ | 1.20x10^3^ |
|  | Pumice stone | 4.80x10^5^ | 7.60x10^5^ | 5.20x10^5^ | 1.25x10^5^ | 1.36x10^5^ | 4.00x10^4^ | 1.48x10^3^ | 1.80x10^3^ | 2.08x10^3^ | 8.00x10^2^ | 8.00x10^2^ | 2.00x10^3^ | 3.20x10^2^ | 1.16x10^3^ | 7.60x10^2^ | 5.60x10^2^ | 3.60x10^2^ | 1.60x10^2^ | 4.00x10^2^ | 4.00x10^2^ | 4.00x10^2^ |
|  | Douglas fir | 3.60x10^4^ | 8.00x10^3^ | 2.40x10^4^ | 1.20x10^2^ | 0 | 2.40x10^2^ | 4.00x10^1^ | 0 | 0 | 0 | 0 | 4.00 | 0 | 0 | 4.00x10^1^ | 0 | 0 | 0 | 0 | 0 | 0 |
|  | Varnished Douglas fir | 0 | 8.00x10^2^ | 5.60x10^3^ | 4.00x10^2^ | 1.72x10^4^ | 4.00x10^2^ | 0 | 8.00x10^2^ | 1.20x10^3^ | 0 | 1.52x10^3^ | 1.20x10^2^ | 7.20x10^1^ | 0 | 1.72x10^2^ | 5.20x10^1^ | 3.16x10^2^ | 2.80x10^1^ | 0 | 4.00x10^0^ | 0 |
| Assay 2 | Stainless steel | 4.00x10^4^ | 1.20x10^5^ | 4.00x10^4^ | 1.84x10^4^ | 2.40x10^3^ | 9.60x10^3^ | 4.00x10^3^ | 4.00x10^3^ | 1.20x10^4^ | 2.40x10^4^ | 1.60x10^4^ | 1.20x10^4^ | 1.28x10^4^ | 3.60x10^3^ | 8.00x10^3^ | 7.60x10^3^ | 9.60x10^3^ | 6.40x10^3^ | 4.80x10^3^ | 6.00x10^3^ | 0 |
|  | Maritime pine | 4.00x10^5^ | 4.00x10^5^ | 0 | 6.00x10^4^ | 9.20x10^4^ | 1.84x10^5^ | 9.60x10^4^ | 4.00x10^4^ | 5.60x10^4^ | 1.00x10^5^ | 1.16x10^5^ | 1.04x10^5^ | 2.40x10^4^ | 1.20x10^4^ | 8.00x10^3^ | 1.20x10^4^ | 1.20x10^4^ | 1.60x10^4^ | 4.00x10^0^ | 8.00x10^0^ | 0 |
| Assay 3 | Douglas fir | 3.44x10^4^ | 4.00x10^2^ | 8.00x10^2^ | 8.00x10^2^ | 0 | 3.72x10^4^ | 0 | 4.00x10^1^ | 3.48x10^3^ | 1.20x10^3^ | 0 | 1.72x10^4^ | 0 | 0 | 1.73x10^3^ | 0 | 0 | 0 | 0 | 4.00x10^0^ | 1.20x10^1^ |
|  | Disinfected Douglas fir | 0 | 0 | 0 | 0 | 0 | 0 | 0 | 0 | 0 | 0 | 0 | 0 | 0 | 0 | 0 | 0 | 0 | 0 | 0 | 0 | 0 |

Table S4: Bacterial count in CFU (triplicate values) of *Klebsiella pneumoniae* recovered from stainless steel, pumice stone, Douglas fir and varnished Douglas fir (Assay 1), on stainless steel and maritime pine (Assay 2), and on Douglas fir and disinfected Douglas fir (Assay 3) over 15 days.

| *Klebsiella pneumoniae*  ATCC 700603 | | CFUs | | | | | | | | | | | | | | | | | | | | |
| --- | --- | --- | --- | --- | --- | --- | --- | --- | --- | --- | --- | --- | --- | --- | --- | --- | --- | --- | --- | --- | --- | --- |
|  |  | D0 | | | D1 | | | D2 | | | D3 | | | D6 | | | D7 | | | D15 | | |
|  | Materials | CFU_1_ | CFU_2_ | CFU_3_ | CFU_1_ | CFU_2_ | CFU_3_ | CFU_1_ | CFU_2_ | CFU_3_ | CFU_1_ | CFU_2_ | CFU_3_ | CFU_1_ | CFU_2_ | CFU_3_ | CFU_1_ | CFU_2_ | CFU_3_ | CFU_1_ | CFU_2_ | CFU_3_ |
| Assay 1 | Stainless steel | 1.32x10^4^ | 1.72x10^4^ | 1.92x10^4^ | 1.20x10^3^ | 0 | 1.20x10^3^ | 4.28x10^2^ | 6.00x10^1^ | 4.84x10^2^ | 3.20x10^1^ | 7.20x10^1^ | 2.28x10^2^ | 1.40x10^2^ | 1.08x10^2^ | 8.00x10^1^ | 1.20x10^1^ | 1.20x10^1^ | 6.40x10^1^ | 5.20x10^1^ | 6.40x10^1^ | 1.28x10^2^ |
|  | Pumice stone | 2.00x10^3^ | 4.00x10^3^ | 1.20x10^3^ | 4.00x10^2^ | 1.20x10^3^ | 4.00x10^2^ | 2.44x10^2^ | 5.80x10^2^ | 1.80x10^2^ | 1.60x10^1^ | 2.80x10^1^ | 8.00x10^0^ | 4.00x10^0^ | 4.00x10^0^ | 8.00x10^0^ | 4.00x10^0^ | 1.20x10^1^ | 8.00x10^0^ | 1.20x10^1^ | 4.00x10^0^ | 4.00x10^0^ |
|  | Douglas fir | 4.00x10^2^ | 4.00x10^2^ | 5.60x10^3^ | 8.00x10^0^ | 0 | 0 | 0 | 0 | 0 | 0 | 0 | 0 | 0 | 0 | 0 | 0 | 0 | 0 | 0 | 0 | 0 |
|  | Varnished Douglas fir | 2.56x10^4^ | 1.28x10^4^ | 1.00x10^4^ | 8.00x10^1^ | 6.80x10^2^ | 2.00x10^2^ | 0 | 7.20x10^1^ | 2.32x10^2^ | 4.00x10^0^ | 4.00x10^0^ | 4.00x10^0^ | 4.00x10^0^ | 0 | 0 | 0 | 0 | 0 | 0 | 0 | 0 |
| Assay 2 | Stainless steel | 1.20x10^4^ | 4.00x10^3^ | 0 | 0 | 1.20x10^3^ | 1.20x10^3^ | 4.00x10^1^ | 4.40x10^2^ | 1.04x10^3^ | 2.80x10^2^ | 2.40x10^2^ | 1.04x10^3^ | 8.00x10^2^ | 0 | 4.00x10^2^ | 4.00x10^1^ | 0 | 1.48x10^3^ | 7.60x10^1^ | 1.60x10^1^ | 0 |
|  | Maritime pine | 1.40x10^4^ | 1.48x10^4^ | 2.63x10^4^ | 8.00x10^2^ | 1.60x10^3^ | 1.20x10^3^ | 1.16x10^2^ | 6.00x10^1^ | 2.36x10^2^ | 6.40x10^1^ | 3.96x10^2^ | 9.60x10^1^ | 1.04x10^2^ | 4.80x10^1^ | 9.20x10^1^ | 0 | 7.20x10^1^ | 2.40x10^1^ | 0 | 0 | 0 |
| Assay 3 | Douglas fir | 1.20x10^2^ | 4.00x10^1^ | 4.40x10^2^ | 3.60x10^1^ | 4.00x10^0^ | 0 | 4.00x10^0^ | 4.00x10^0^ | 0 | 0 | 0 | 0 | 0 | 0 | 0 | 0 | 0 | 0 | 0 | 0 | 0 |
|  | Disinfected Douglas fir | 0 | 0 | 0 | 0 | 0 | 0 | 0 | 0 | 0 | 0 | 0 | 0 | 0 | 0 | 0 | 0 | 0 | 0 | 0 | 0 | 0 |
